# Supplementary material for: Optimization and inventory management under stochastic demand using metaheuristic algorithm
Source: PLoS One. 2024 Jan 5;19(1):e0286433. doi: 10.1371/journal.pone.0286433 (PMC10769039; doi:10.1371/journal.pone.0286433)
Supplement: Attachment — Submitted filename: 23Plone_TanAA_QA_Fnn.docx [file pone.0286433.s001.docx]

**Reviewer’ Comment and Author’ Reply**

**(MS ID:** PONE-D-23-01150**)**

The revised version of the paper is submitted to the ***PLOS ONE***

**Manuscript Title**: *Optimization and inventory management under stochastic demand using meta*heuristic algorithm

=====================================================================

**Point-By-Point Responses**

All revisions are made according to valuable comments from editors and reviewers. Newly
added sentences and all corrections are written in red color in the revised manuscript. We have
checked the whole manuscript and made all necessary changes. Also, we have done our best on reediting for grammatical correctness as suggested. We are submitting the corrected manuscript with the suggestion incorporated into the revised manuscript. Further explanations on major revisions are now described below.

--------------------------------------------------------------------------------------------------------------------
**Responses to Reviewers' comments**

**Review #1.**

*Thank you for your thoughtful, helpful, and kind review of the manuscript. Your comments and suggestions have been incorporated as appropriate into the revised draft. Specific revisions are noted below.*

1. The abstract section is fragile. Please re-write an abstract section, explain an obtained result and contribution, improve a proposed method, etc. Please delete unnecessary information.

**Answer**: Thank you for your comment. We rewrite an abstract section focusing on the contribution, methods, and result. Also the unnecessary information has been deleted in the revised manuscript.

2. This paper has more than spelling and grammatical errors. Please fix all of them.

**Answer**: As the reviewer pointed out, we have checked the typos and grammatical mistakes. Additionally, the manuscript has been revised according to the professional editing service by Editage (www.editage.co.kr).

3. The authors should clearly state the limitations of the proposed method in other applications.

**Answer**: We have provided some citations on the applications and its possible limitations of this method. Please have a look at the first paragraph Section 2.

4. There are many multi-objective GWO algorithms in the literature, what is the difference between your algorithm with theirs?

**Answer:** According to reviewer comments, we have provided further comparisons between our algorithm and other existing works. Among swarm intelligence (SI) algorithms in metaheuristic framework, we implement intelligence paradigms focusing on low-computational yet high-performance strategies for solving sophisticated optimization problems. Its ease of use and flexibility have fueled its adoption in the inventory management. We realize that GWO would best suit our needs and is the most prevalent SI-based optimization algorithm: it is simple to implement and has a reasonable runtime speed. Compared to a Genetic Algorithm (GA), GWO has a much lower computational cost because it does not have genetic operators such as mutation or crossover as GA does, which can expand the search space significantly. Similarly, Ant Colony Optimization (ACO) is mainly used in scheduling or routing problems where the termination condition is predetermined. Some other swarm-based heuristics, such as Particle Swarm Optimization (PSO), Firefly Algorithm (FA), or Cuckoo Search Algorithm (CSA), have more complicated searching mechanics, which would increase complexity unnecessarily. To summarize, key strengths are that the update process in each iteration is less complicated, and GWO has fewer parameters to be tuned compared to other swarm-based heuristics. Particularly, the MOGWO approach best fits inventory management for multi-objective optimization problems.

5. Please add a flowchart of the proposed method.

**Answer**: For providing a holistic understanding of the proposed research for the reader, an illustrative overview of the MOGWO approach has been added in the revised manuscript (Figure 4)

6. The related work section is missing; please add this section. And make the Introduction and related work sections more productive using the following articles. Reading and using these articles:
   o <https://doi.org/10.1007/s00354-022-00188-w>
   o <https://doi.org/10.1007/s42235-022-00288-9>
   o <https://doi.org/10.1007/s00366-021-01369-9>

References are not up to date and not enough. Please improve this item.

**Answer:** Thank you for your great comments. We have cited these articles to update our references and make our introduction more abundant. The introduction has been divided into two separate sections for more detailed information and better readability: Section 1 (Introduction) and Section 2 (The related work).

7. How did the authors set parameters for your proposed algorithm? Please make sensitivities of these parameters to the performance of your proposed algorithm!

**Answer:** To answer how the parameters are set up, first we would like to summarize process below. In general, they were determined by after several experiments and tests, and we adjusted the parameters for more optimal results. They are typically values in supply chain management. Please refer to the parameter in Table 2 of Section 4, where there are mean, standard deviation of demand, purchase cost, order cost, holding cost and other parameter have been list. For stochastic parameters, they demand (Di,j), item’s probability (Poi,j) and the size or the quantity of item (Soi,j), they are stochastic or random values and created by using some random function as the log-normal distribution (to provide Poi,j and other function to make the item’s size random), from that the demand is also stochastic. This parameter is explained by Equations (2), (3) and (4) and is calculated based on the number, figures in table 2. For decision parameters, they are quantity order (Q) and the reorder point (r), which means they will affect directly to the objective functions as the revenue and the storage space. Therefore, Table 3 has shown the sensitive analysis of Q and r, to see how they affect our algorithm’s result. We also offer sensitivity analysis and show that the optimized strategy works well even when used in settings with some varied functions.

8. All the structural problems in this study are very popular, therefore the authors suggest comparing with results obtained from previous studies to convince the power of the proposed algorithm. Moreover, these structural problems are very easy, please try to solve other complex problems.

**Answer:** Due to the novelty GWO, there is a few studies in the literature to design a multi-objective version of this algorithm. First, to compare with results obtained from previous studies, here two key findings for MOGWO are described as follows:

- MOGWO might be considered as one of the best optimal algorithms currently when compared to other algorithms, such as Multi-Objective Water Cycle Algorithm (MOWCA), Multi-objective Particle Swarm Optimization (MOPSO) and Non-Dominated Sorting Genetic Algorithm- II (NSGA-II). This observation is proved by Maadanpour et al, (2021), and a tri-objective mathematical model is proposed for the Transportation-Location-Routing problem. Then four performance measures are employed to conclude that MOGWO is ranked as the first and best algorithm in solving proposed tri-objective mathematical model.
- Another research presented some comparisons between MOGWO and NSGA-II in Green two-echelon closed and open location-routing problem (Heidari et al., 2022). Their results also show that in general, MOGWO algorithm has a better performance than the NSGAII algorithm.

Second, the structural problems are described by

- We use a lognormal distribution to create a variable which stands for a probability of an item. This probability means there will be *x*% that a customer will order that item in any given day in period T, and in case it is ordered, the number size of order is also a random function. Basically, each item has different demands, one item could be usually ordered in period T, and some items are just ordered in some periods or not continuously.
- From the demand, we use a function to simulate inventory behaviors and then calculate the profit. The simulation in this research is not presented completely, we also apply Monte Carlo method to simulate the inventory behaviors. More updated results have been presented to show how we could implement these methods.

Therefore, we believe that the proposed method is very powerful for solving multi-objective optimization problems.

9. The authors should provide the code of your proposed algorithm.

**Answer:** For considering limited space provided, the pseudocode of the proposed MOGWO is only given in the manuscript. Our actual code is just based on the algorithm with pseudocode, written by Python.

10. Please write a contribution to your paper in the Introduction section.

**Answer:** Thank you for your comment. We have presented the novelty and major contribution of this study in the revised manuscript.

11. Please change the title of the end section (Conclusion) to (Conclusion and Future Works), and write some future works.

**Answer:** As requested, we have changed the titles of some sections. We have
checked the whole manuscript and made all necessary changes.

12. Please use a new comparison algorithm, such as the Farmland fertility algorithm, African Vultures Optimization Algorithm, Mountain Gazelle Optimizer, and Artificial Gorilla Troops Optimizer.

**Answer:** Based on complete literature review, we have provided general comparisons on various meta-heuristic approaches. Please refer to question 4 and question 8 for comparisons. Our algorithm has been implemented by incorporating pros and cons of other algorithms you mentioned.

13. Expand the critical results in the conclusion. Focus on the main developments in the finale. Also, write the main contributions in the conclusion.

**Answer**: Along with the questions 10 and 11, we have also expanded the critical results and main developments in the revised manuscript.

14. Numerical results are good enough, but more explanations are required to analyze each figure presented.

**Answer:** As recommended, we have checked again all the figures and their explanations. We have provided necessary explanations on the results.

15. All figures have low quality, and please improve all of them.

**Answer**: As requested, we provide the image files with high quality.

**Review # 2.**

*Thank you for your thoughtful, helpful, and kind review of the manuscript. Your comments and suggestions have been incorporated as appropriate into the revised draft. Specific revisions are noted below.*

1. The novelty and findings of this study are not stated in the Abstract, and it is recommended to rewrite it.

**Answer:** Thank you very much for your valuable comments. As requested, we have rewritten the abstract by highlighting novelty and key findings.

2. The English language needs significant revision.

**Answer:** We have checked the whole manuscript and made all necessary changes. Additionally, the manuscript has been revised according to the professional language editing service by Editage (www.editage.co.kr).

3. The title cannot reflect this problem and novelty, and it is recommended to revise it.

**Answer:** Thank you for your comment. We have revised the titles of the sections to reflect this optimization problem.

4. The contributions of this study should be summarized at the end of the Introduction.

**Answer:** Thank you for your comment. We have presented the novelty and major contribution of this study in the revised manuscript.

5. This study suffers from a lack of an in-depth literature review on the problem of this study and recent metaheuristic algorithms through which reviewing the following recent algorithms are recommended. Quantum-based avian navigation optimizer algorithm, An improved moth-flame optimization algorithm with adaptation mechanism to solve numerical and mechanical engineering problems, DMDE: Diversity-maintained multi-trial vector differential evolution algorithm for non-decomposition large-scale global optimization, Migration-based moth-flame optimization algorithm, and Starling murmuration optimizer: A novel bio-inspired algorithm for global and engineering optimization.

**Answer**: As requested, we have provided an in-depth literature review on the related topics in introduction section. This extensive review aims to highlight the research gap between our work and the others in the supply chain literature.

6. The nomenclature table is recommended for the parameters used in all equations.

**Answer:** According to reviewer’s comments, we have provided the nomenclature table (Table 1) for the parameters used in all equations.

7. The importance of this study is not clear why the new multi-objective version of GWO is proposed.

**Answer:** Among swarm intelligence (SI) algorithms, we focus on low-computational yet high-performance algorithms to estimate the model weights and solve real-world problems. So far, there are only a few studies in the literature implementing a multi-objective version of this algorithm. We realize that GWO would best meet our needs and is the most prevalent SI-based optimization algorithm: it is simple to implement and has a reasonable runtime speed. In this research, a new type of multi-objective version of GWO is proposed for optimization methods to inventory management problem by considering profit and storage space. To summarize, key strengths are that the update process in each iteration is less complicated, and MOGWO has fewer parameters to be tuned.

8. Please check the title "3. Multi-objective optimization using grey wolf optimizer".

**Answer:** We have checked the title of Section 3. For clarity, the title is now changed to Multi-objective optimization using GWO algorithm.

9. This study's novelty is unclear, and it is suggested to consider the proposed method section and explain your novelty clearly.

**Answer:** We have rewritten the introduction section to explain the novelty for clarity. In addition, the proposed method is described in Section 3, which deals with MOGWO for inventory optimization.

10. The claims of this study should be stated, and the authors explain which experimental evaluation can support their claims.

**Answer:** There are only few studies devoted to the design of efficient multi-objective optimization algorithms for managing supply chain networks. This study introduces a new approach to solve the inventory management problem which is realized using MOGWO to optimize objective functions of profit and storage space. Both objective functions are the outputs of an inventory model affected by stochastics demand which is highly unpredictable. Figure 5 shows the random demand for each item, and our algorithm could provide the decision support system for finding the optimal solution under stochastic demand.

11. It is recommended to check the pseudocode of the multi-objective grey wolf optimizer (MOGWO).

**Answer:** Thank you for your careful review, we have check and rewrite some contents in the pseudocode of the multi-objective grey wolf optimizer (MOGWO) to bring the algorithm up to the highest standards.

12. It is recommended to compare MOGWO with other multi-objective algorithms.

**Answer**: According to reviewer recommendation, we have provided a comprehensive review on multi-objective algorithms.

13. To compare with results obtained from previous studies here are two other studies that also prove that MOGWO has better results by some conditions.

**Answer:** MOGWO can be considered as one of the best optimal algorithms currently when compared to other algorithms such as Multi-Objective Water Cycle Algorithm (MOWCA), Multi-objective Particle Swarm Optimization (MOPSO) and Non-Dominated Sorting Genetic Algorithm- II (NSGA-II). This observation is proved by Maadanpour et al, (2021), they proposed a tri-objective model for the Transportation-Location-Routing problem and implemented all above algorithms. Then four performance measures are introduced to conclude that MOGWO is demonstrated as the best algorithm in solving proposed tri-objective model. Another research has performed comprehensive comparisons between MOGWO and NSGA-II in Green two-echelon closed and open location-routing problem (Heidari et al., 2022). Their results also show that MOGWO algorithm provides a better performance than the NSGAII algorithm.

14. The Conclusion should be extended to include more details regarding the limitations of the proposed study.

**Answer:** Thank you for valuable comments. Conclusion section has been redrafted for clarity, including the future research directions and limitations.

15. The nonparametric statistical test is recommended.

**Answer:** The statistical test is also provided in Table 2, where the decision variable has been changed to see performance difference of both objective functions.
