# Supplementary material for: Optimization and inventory management under stochastic demand using metaheuristic algorithm
Source: PLoS One. 2024 Jan 5;19(1):e0286433. doi: 10.1371/journal.pone.0286433 (PMC10769039; doi:10.1371/journal.pone.0286433)
Supplement: Attachment — Submitted filename: rre23Plone_TnYs_QA_Fnnn.docx [file pone.0286433.s002.docx]

**Reviewer’ Comment and Author’ Reply**

**(MS ID:** PONE-D-23-01150R1**)**

2^nd^ revised version of the paper is submitted to the ***PLOS ONE***

**Manuscript Title**: *Optimization and inventory management under stochastic demand using metaheuristic* *algorithm*

=====================================================================

**Point-By-Point Responses**

All revisions are made according to valuable comments from editors and reviewers. Newly
added sentences, and all corrections are written in red color in the revised manuscript. We have
checked the whole manuscript and made all necessary changes. Also, as suggested, we have done our best on reediting for grammatical correctness. We are submitting the corrected manuscript with the suggestion incorporated into the revised manuscript. Further explanations on major revisions are now described below.

--------------------------------------------------------------------------------------------------------------------

**Reviewer #1:**

From the response letter, I think the paper has been well revised according to the previous reviewers, and the current version of the manuscript is acceptable for publication.

Author response:

*Thank you for your thoughtful, helpful, and kind review of the manuscript. Your comments and suggestions have been incorporated as appropriate into the revised draft.*

**Reviewer #2:**

Thank the authors for responding to some of the comments; however, they have not responded to some of the suggested comments adequately. I recommend authors address the following issues before accepting for publication.

**Author response**:

*Thank you for your thoughtful, helpful, and kind review of the manuscript. Your comments and suggestions have been incorporated as appropriate into the revised draft. Specific revisions are noted below.*

1. It is recommended to revise a paper based on the journal's guidelines. The style of references should be revised, and some sentences and abbreviations of the algorithm need references. Please check the following paragraph. "Lately, the following approaches have attracted extensive attention in solving various problems owing to their…"

**Author response**: According to the reviewer’s comment, we have revised the paper based on the journal's guidelines. Also, we have checked and revised the paragraph mentioned.

2. The related work still can be improved by adding more recent algorithms as well as their applications.

**Author response**: As requested, we have provided more information on the related works by introducing new optimization algorithms and their applications (Subsection 1.1).

3. In Fig. 4, the arrows of boxes t+1 and the calculated fitness value should be rechecked.

**Author response**: Thank you for your kind review of the manuscript. The arrows of boxes “*t* + 1” and “ the calculated fitness value” have been updated, so their arrows are clearly separated in the figure. Please check the updated Fig 4 for clarity.

4. The description of equations 15 and 16 should be revised.

**Author response**: According to the reviewer’s comment, we have improved the description of equations (15 and 16).

5. The claims of this study should be stated, and the authors explain which experimental evaluation can support their claims.

**Author response**: The claim and the experimental evaluation have been updated and marked clearly in the Abstract and Conclusion sections.
